# Hypokalemia induced rhabdomyolysis

**DOI:** 10.4103/0971-4065.78085

**Published:** 2011

**Authors:** V. V. Jain, O. P. Gupta, S. U. Jajoo, B. Khiangate

**Affiliations:** Department of Medicine, Mahatma Gandhi Institute of Medical Sciences, Sewagram, Wardha, India

Sir,

Rhabdomyolysis is usually associated with hyperkalemia due to renal failure and hence hypokalemia as a cause of rhabdomyolysis is usually missed.[1] We want to highlight the association of hypokalemia with rhabdomyolysis.

An 18-year-old unmarried girl presented with a history of pain in the abdomen and nonbilious vomiting of 4-day duration. This was followed by painful muscle cramps and progressive proximal muscle weakness involving all four limbs. She had no history of diarrhea, diuretic intake, fever, arthralgia, and rash. She had a similar episode of abdominal pain, vomiting, and muscle weakness 2 months prior to this illness which recovered after receiving some IV injections in a private hospital. She had never passed any urine stones. She was normotensive and nondiabetic with a normal thyroid profile. There was no history of drug intake.

On examination she had a blood pressure of 110/70 mm of Hg. The muscle power was 3/5 in proximal muscle groups of all four limbs with flaccidity and depressed deep tendon reflexes. There was no bulbar weakness, no cranial nerves involvement, and no sphincter involvement. Plantar response was flexor. There were no fasciculation, myoclonus, or muscular atrophy.

Investigation showed hypokalemia (1.6 mEq/L), markedly raised creatine phosphokinase (CPK) (7960 IU/L), and metabolic acidosis with high serum lactate. Her serum creatinine was 1.2 mg/dL and serum sodium was 138 mEq/L. The urinanalysis was normal. Urine K^+^was 5 mEq, suggestive of hypokalemia due to gastrointestinal loss. She was diagnosed as a case of hypokalemia causing rhabdomyolysis. She was treated with fluid replacement and IV potassium replacement. After 10 days, the muscle weakness recovered following a rise in serum potassium. On discharge, her CPK had decreased to 514 IU/L which normalized after 3 weeks.

Our patient had a history of persistent vomiting which led to hypokalemia due to gastrointestinal loss, and urine potassium was low (5 mEq/L). She was normotensive and did not have sodium conservation or features of primary aldosteronism. The renal cause of hypokalemia was also ruled out on the basis of low urinary potassium. Though she had metabolic acidosis, it could have been due to high serum lactate because of rhabdomyolysis itself.

In the literature, there are few documented cases of hypokalemia due to primary aldosteronism[2] leading to rhabdomyolysis. Licorice ingestion,[3] abuse of laxatives, proximal renal tubular acidosis, and diuretics have also been associated with hypokalemia-induced rhabdomyolysis. There are no reports in the literature on hypokalemia due to vomiting leading to rhabdomyolysis.

Potassium plays a major role in regulating the skeletal muscle blood flow; an increased potassium concentration in the muscle during muscle activity causes vasodilatation, which increases the regional blood flow.[4] In state of hypokalemia, this increase is hampered causing relative ischemia in the active muscle consecutively leading to muscle cramps, and in a severely depleted state may cause muscle necrosis and rhabdomyolysis.[4] In addition to hypoperfusion, hypokalemia-induced impairment in muscle metabolism also may contribute to muscle dysfunction.[5]
